# Ortho-para interconversion of nuclear states of H_2_O through replica transition state: prospect of quantum entanglement at homodromic Bjerrum defect site

**DOI:** 10.1007/s00894-023-05646-w

**Published:** 2023-07-12

**Authors:** Sanyasi Sitha

**Affiliations:** Department of Chemical Sciences, APK Campus, University of Johannesburg, PO Box 524, Auckland Park, Johannesburg, 2006 South Africa

**Keywords:** Water dimer, Bjerrum defect, Ortho-para interconversion, Homodromic, Entanglements

## Abstract

**Context:**

From a nuclear spin prospective, water exists as *para* and *ortho* nuclear spin isomers (isotopomers). Spin interconversions in isolated molecules of water are forbidden, but many recent reports have shown them to happen in bulk, through dynamic proton exchanges happening between interconnected networks of a large array of water molecules. In this contribution, a possible explanation for an unexpected slow or delayed interconversion of ortho-para water in ice observed in an earlier reported experiment is provided. Using the results of quantum mechanical investigations, we have discussed the roles played by Bjerrum defects in the dynamic proton exchanges and *ortho-para* spin state interconversions. We guess that at the sites of the Bjerrum defects, there are possibilities of quantum entanglements of states, through pairwise interactions. Based on the perfectly correlated exchange happening via a replica transition state, we speculate that it can have significant influences on *ortho-para* interconversions of water. We also conjecture that the overall ortho-para interconversion is not a continuous process, rather can be imagined to be happening serendipitously, but within the boundary of the rules of quantum mechanics.

**Methods:**

All computations were performed with Gaussian 09 program. B3LYP/6-31++G(d,p) methodology was used to compute all the stationary points. Further energy corrections were computed using CCSD(T)/aug-cc-pVTZ methodology. Intrinsic reaction coordinate (IRC) path computations were carried out for the transition states.

## Introduction

**Fig. 1:**
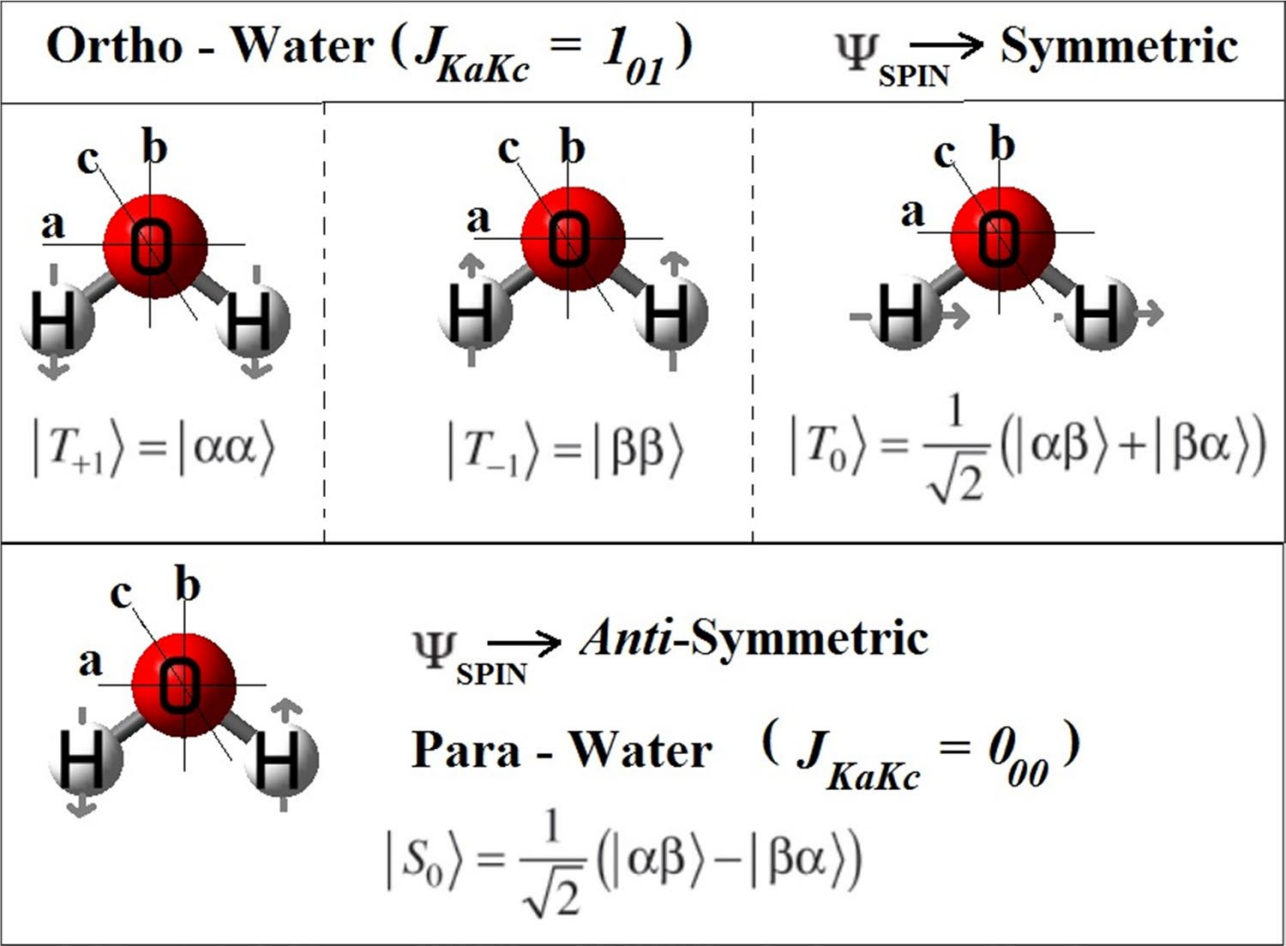
Nuclear spin isomers (isotopomers) of water. In the electronic and vibrational ground, ortho-water exists in a single *J*_*KaKc*_ = 1_01_ rotational state, whereas the para-water exists in three different *J*_*KaKc*_ = 0_00_ rotational states. [2, 3] Here, *J* represents the quantum number of total rotational angular momentum, and *K*_*a*_ and *K*_*c*_ are related to the projections of *J* onto the principal axes of inertia, *a* and *c*, respectively

Water molecules are known to exist as two nuclear-spin isomers (isotopomers). In those nuclear-spin isomers, two distinct alternative orientations of the nuclear spins of the two hydrogen atoms result in *ortho-* and *para-*waters. [1] When the nuclear spins of the two hydrogens are parallel (total nuclear spin: *I* = 1); then, it is called as *ortho*-H_2_O (a nuclear triplet state, |*T*_*k*_⟩, where *k* = −1, 0, and +1), and when the spins are anti-parallel (total nuclear spin: *I* = 0); then, it is called as *para*-H_2_O (a nuclear singlet state: |*S*_0_⟩). [2] Applying Dirac’s coupled two spin ½ particles (fermion) in a system theory, in the case of water, one can get four lowest eigen states (pictorial representations, with their corresponding spin states with asymmetric top notations are shown in Fig. 1). [3] This difference in *I* also gets manifested in their nuclear statistical weight, resulting an ortho (2*I* + 1): para number density ratio as 3:1, in the elevated temperature conditions. [3, 4]

It is well known that nuclear-spin isomeric interconversions between *ortho* and *para* isotopomers in isolated water molecules are forbidden. [2, 4] Hence, any perturbation, like collisions or electromagnetic radiations or even chemical reactions, cannot violate the nuclear-spin symmetry conservation in water molecules. [2, 4] On the other hand, many recent reports demonstrated the nuclear-spin-symmetry interconversions and isomer enrichments through interconnected networks of a large array of water molecules, as present either in the liquid water or ice. [4–29] In one such report by Tikhonov and Volkov, published in the journal Science, they mentioned “The relaxation time needed to convert spin-modified ice to the 3:1 equilibrium state was estimated to be at least a few months long. [4]” Based on the rates of proton exchanges for ice, they mentioned such behavior to be highly unexpected and also indicated that the reasons are unclear. [4] They concluded that “Fast proton exchange obviously does not lead to the fast Ortho-Para (OP) conversion. The exchange without OP transitions, i.e., without change of energy (of resonance character), distinctly dominates.” [4].

It is well-known that the proton exchanges happening within a single molecule, and/or between two or more molecules, can be regarded as quantum exchanges of states, occurring through the exchanges of nuclear spin moments, within a single molecule or between the molecules, respectively. [3] Thus, one can say that the rapid and the dynamic proton exchanges existing either in the liquid water or ice can play important roles in the formations as well as conservations of the observed spin isomers of water. [30] It is well known that for a molecule to show the nuclear spin isomers (like H_2_O), it needs to satisfy one necessary condition of the chemical (and symmetric) equivalence, with respect to the positions of the nuclei responsible for the nuclear spin isomers. [16, 31] In light of this chemical equivalence principle, we speculate that at the site where the quantum exchanges of states (say between two molecules) are happening, not only the exchanges of nuclear spin moments play important roles but also a symmetric nature of the exchanges (or chemically equivalence nature of exchanges, with a double proton exchange) can possibly create quantum entanglement of states of the two participating molecules. As indicated by earlier researchers, for a possible quantum entanglement, replica (or a π-rotational symmetric) like chemical equivalence at the site of exchange (or at the transition state) can be regarded as a necessary condition. [31].

Again, statements of Tikhonov and Volkov (*vide supra*) clearly advocate that selective proton exchanges between water molecules play very important roles for the delayed ortho-para conversions. [4] We guess that the selective nature affecting the slower conversion of ortho-para might be influenced by the possibilities of quantum entanglements. To propose a logical answer to the abovementioned unusual behavior and roles of proton exchanges, we revisited the chemistry of water dimer (numerous theoretical and experimental reports can be found in the literature. Only selected few references are provided here [32–54]**)**, using quantum mechanical computations. The motivations for this work are inspired by some recent experimental works on STM tracking of the dynamic proton exchange processes happening at Bjerrum D-type defect sites in water ice. [55–60] Based on our completely new insightful observations from these recent experimental findings, we conjectured that the Bjerrum defect (which breaks the ice rule) can facilitate possible quantum entanglement via a replica transition state (symmetric and chemically equivalent). In our view (purely qualitative in nature), the Bjerrum defect sites in a way may be acting as the regulators for the rapid dynamic proton exchanges happening in the ice lattice by providing the platform for equally probable ortho-to-para and para-to-ortho interconversions and a possible quantum engagement between two water molecules.

## Computational methods

All computations were performed with the implemented methods in Gaussian 09. [61] All the stationary points reported in this work were fully optimized using B3LYP [62, 63] methodology, and then the energy corrections were carried out using CCSD(T) [64, 65] methodology on those optimized geometries. As this work is related to revisiting the water dimer chemistries to find some interesting insights from various other assessments; hence, computing the accurate energies was not the main aim. To address this aspect, great deals of works exist in the literature with more detailed investigations with accurate energetics (with various quantum mechanical methodologies). This work is more focused on giving some rational explanations to a previously reported unusual experimental observation. While for the B3LYP methodology, 6-31++G(d,p) basis set was used; at the same time, energy corrections were carried out at CCSD(T)/aug-cc-pVTZ level of theory. From the computed vibrational frequencies, true local minima (all positive frequencies) and the transition states (one imaginary frequency or negative eigen value in the Hessian) were established. Transition states were further confirmed from the intrinsic reaction coordinate (IRC) path computations. [66].

## Results and discussion

### Ice rules and Bjerrum defects

**Fig. 2:**
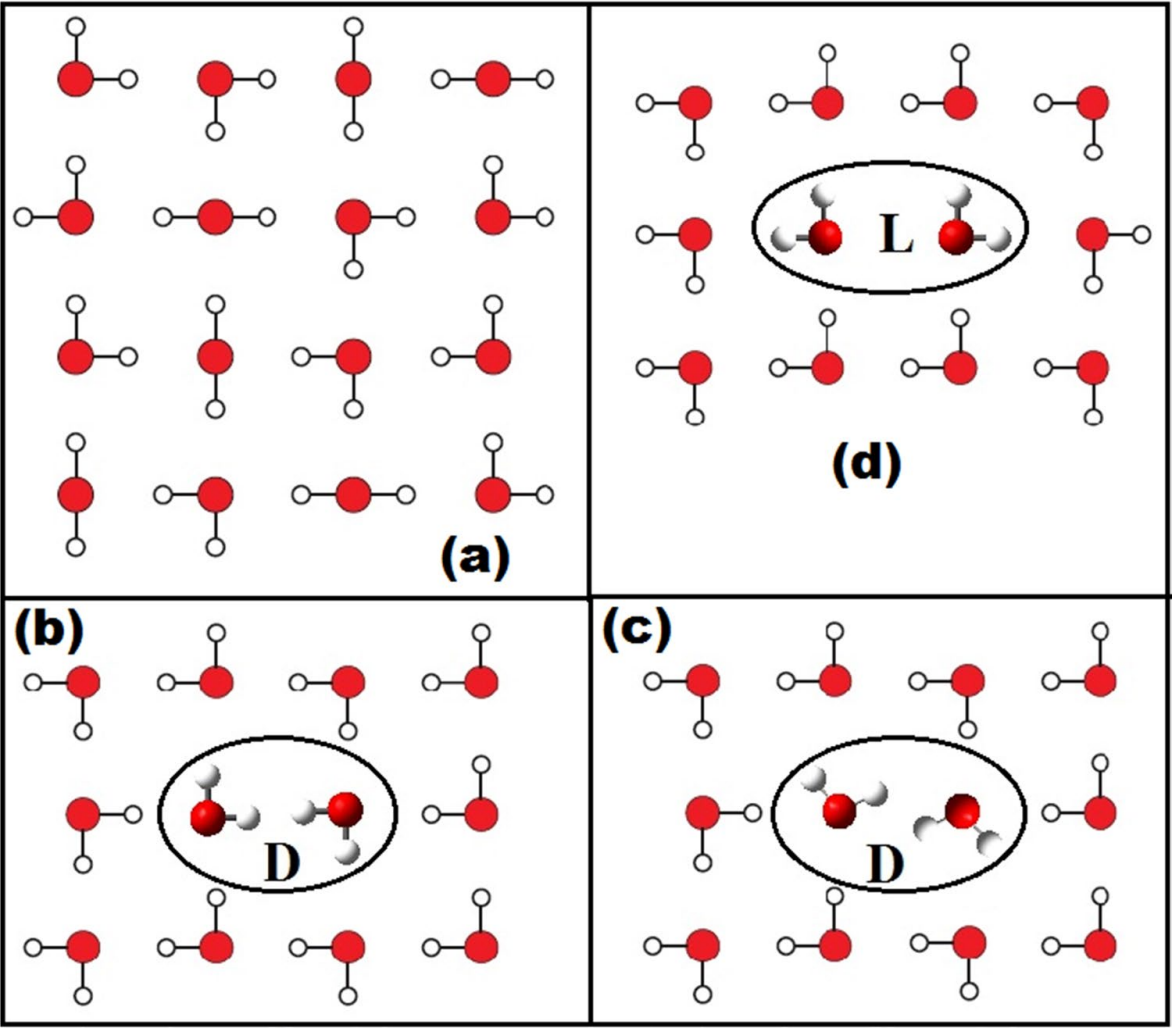
Two-dimensional (2D) schematic representations of *Ice Ih* lattice (red represents oxygen, and white represents hydrogen): **a** defect free ice lattice, **b** Bjerrum D-defect site, **c** a more realistic view of D-defect site, and **d** Bjerrum L-defect site

Ice is known to exist various structural forms. [67–72] *Ice Ih*, which exhibits hexagonal structure, is well known as well as most common in nature. [68] In the crystal structures of *Ice Ih* or in general in most of the known ice structures, orientations of the water molecules are not random, rather each molecule of water is surrounded by four other nearby water molecules, located at the four corners of a tetrahedron. [71] In this fulfilled primary requirement, crystal structure of ice follows two more important points of the Bernal-Fowler ice rules. [73] Rule 1: Each molecule of water simultaneously acts as an acceptor for two hydrogen atoms (forms two hydrogen bonds as an acceptor) from two immediate neighboring water molecules and also at the same time acts as a donor for two hydrogen atoms (forms two hydrogen bonds as donor) to the two other nearest-neighboring water molecules. [73] In a simpler term, water like a human has two hands (two hydrogens: say H stands hand) and two legs (two lone pairs: say L stands for leg), with oxygen atom as the controlling head. Rule 2: There is only one hydrogen atom shared between each pair of oxygen atoms (while covalently bonded to one O-atom, at the same time hydrogen bonded to other). In a simpler word, these two rules put a limit to the extent of sharing. [73] A two-dimensional representation illustrating the above two rules is shown in Fig. 2(a). [71]

Crystal structures of ice are not defect free, rather violations of the above two rules are known to produce two different types of defects. [72] In the first kind of defect, which violates the rule 1 (*vide supra*), ionic defect pairs are formed in the lattice (not the focus of this work). [55–60, 72] Second kind of defect is commonly known as the Bjerrum defect and arises due to the violation of the rule 2. [55–60, 72–74] Bjerrum defect arises due to the rotation of a water molecule and thus can be created by simple rotation of one water molecule in the crystal lattice. Such a rotation can lead to two possibilities (shown in Fig. 2 as representative 2D crystal lattices). [74] In one case, where the space between two O-atoms is crowded by two hydrogen atoms (a Bjerrum D-defect: Fig. 2(b) and (c)) and whereas in the other case, the space between two O-atoms is completely devoid of any hydrogen atom (a Bjerrum L-defect: Fig. 2(d)). Between 2(b) and 2(c) shown for the D-defects, the former one is an ideal 2D schematic representation, while the later one is a realistic view. [72–74] Actually, due to strong electrostatic interaction between the two closely placed and covalently bonded (different O-atoms) protons, they tend to repel each other, resulting a situation as represented in Fig. 2(c). [55] At the site of defects, for a dynamic proton exchange to happen, Bjerrum L-defects are definitely and can be regarded as not suitable. [55] Hence, further discussions in this work are focused on Bjerrum D-defects only, as such a defect can be believed to be capable of a dynamic double proton exchange. [55] Worth to mention here is that at the Bjerrum D-defect sites, apart from the two hydrogen atoms which can render dynamic proton exchanges, the remaining two hydrogens (free hydrogens) that lie alternatively, one above and one below the plane of the two oxygen atoms of the water dimer, can result in a frustrated structure of the ice (or the water dimer) [55].

### Replica conformations for Bjerrum defects

**Fig. 3:**
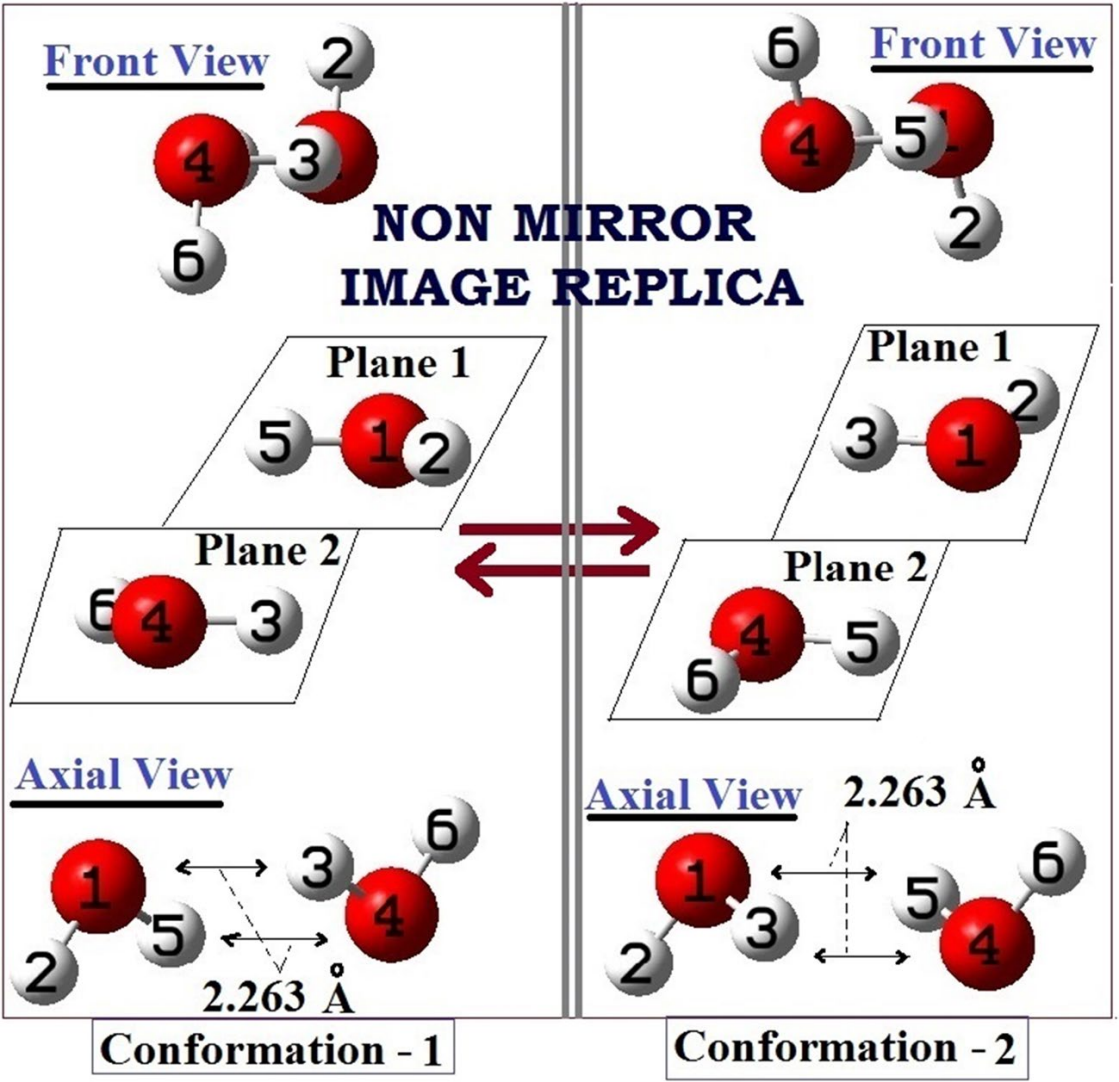
Pictorial representations of dynamic double proton exchanges (donor-acceptor exchange) between nuclear spin isomers (isotopomers) of (H_2_O)_2_, in the two non-planar equivalent cyclic conformations, from B3LYP/6-31++G(d,p) optimizations

Existences of dynamic double proton exchanges at the sites of Bjerrum D-defects have been shown experimentally (through simulated STM images) by various researchers in the recent times. [55–60] All these experimental reports are pointing out the existences of the cyclic (or ring) conformer (complex) of water dimer (two equivalent conformers are shown as conformer 1 and 2 in Fig. 3). This conformation was never given much importance in all earlier studies related to water dimer. In fact, such a conformer can be regarded as a disobedient entity to Bernal-Fowler ice rules, which governs the structure and patterns of ice lattice. [73] Even earlier computational studies on the PES of the (H_2_O)_2_ clearly indicated the non-planar cyclic conformation to be a metastable conformer with one imaginary frequency. This associated issue was the main cause for not putting much importance to this conformer in the earlier studies related to water dimer. More detailed information about structures and related geometric parameters for the cyclic isomer of water-dimer is not discussed here. More accurate energetics and detailed discussions can be found in many earlier works. [75–86]

The important point to mention about this non-planar cyclic dimer is that it exists in two equivalent non-mirror image replica types of forms. The finding reported here is also strongly supported by few recent experiments, [55–60] where such a non-mirror image replica type of transformation of non-planar cyclic dimer is being reported (observed one conformer before and one conformer after double proton transfer). Worth to mention here that these two forms are non-mirror images (even non-superimposable) of each other, and any symmetry operation cannot convert one conformer to other. But, as can be seen from the Fig. 3, though from the symmetry operations points-of-views they show non-indifference behaviors, but from the structural points-of-views, they are exact structural replica of one another (hence, show degeneracy). Thus, they were also found to be isoenergetic (energetically equal) here. Also, to mention here is that, in those recent experimental reports, a transition state connecting these two replicas (one before and one after the double proton transfer) is being reported. [55–60] From these reported experimental works, two important findings can be extracted: (i) the metastable hydrogen bonded water dimer (two equivalent and replica conformations shown in Fig. 3) exists at the defect sites and responsible for an efficient donor-acceptor exchange and (ii) a transition state interconnecting these two metastable conformations is responsible for the above exchange process. [55–60] As such, a transition state interconnects the conversion as well as dynamic proton exchange path between the two replicas; [55] hence, we can call it here as a “Replica Transition State.”

### Misrepresented replica transition states

**Fig. 4:**
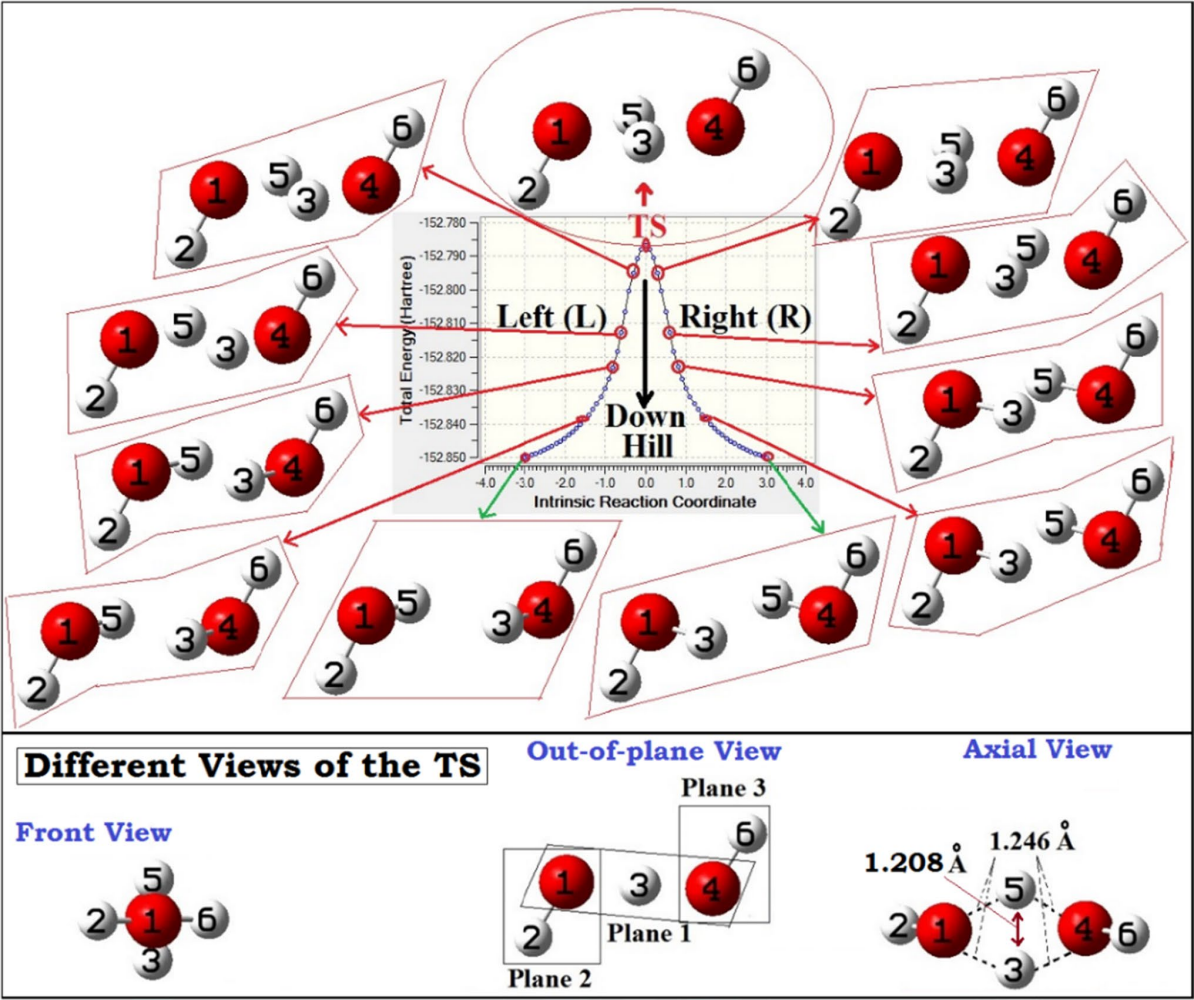
Mapped intrinsic reaction coordinates (IRC) paths for *trans*-TS, computed using B3LYP/6-31++G(d,p) methodology. Sequential structural changes moving downhill from the transition state are also shown from point-to-point (diagram is not to scale)

We tried to locate such a replica transition state for this study, and the computed IRC path of this replica transition state is shown in Fig. 4. Obtained transition state was found to be 47.8 kcal.mol^-1^ above the both isoenergetic replica conformations with the CCSD(T)/aug-cc-pVTZ energies. As mentioned in the previous section, existence of such a replica transition state and responsible for the dynamic double proton exchange process at the Bjerrum D-site is already reported in few recent experiments. [55–60] It is to be noted here that the obtained replica transition state (Fig. 4) was found to be exactly like those earlier reports and comparison of the structural data agrees well with experimental reports. [55] From the recent experimental findings, it was clear that the prior views on this transition state by many earlier reports are not exactly true. In many earlier works, this replica transition state was reported. But, instead of a replica transition state, it is described (we can say rather misrepresented) as a transition state responsible for the donor-acceptor exchange between two singly-hydrogen bonded water molecules (global minimum of water dimer which strictly obeys the Bernal-Fowler ice rules). [75–87] With a thorough analysis, instead, our observations say that it is rather a transition state that represents the proton exchanges happening at the Bjerrum D-defect sites (that defies the Bernal-Fowler ice rules) of an ice lattice. [55]

As it is apparently clear now that the transition state (Fig. 4) is indeed responsible for the double proton exchange process happening at the D-defect sites, [55] now, we analyzed the nature of transition state. From the three different views of the transition state shown in Fig. 4 (bottom section), the mirror symmetric nature of the two hydrogens participating in the double proton exchange process can be seen. Not only that a mirroring type of entanglement but also perfect synchronization in motions of the two particles (hydrogens) during the exchange process were observed. As discussed previously, one necessary condition for a quantum entanglement of states of the two participating molecules is the symmetric nature (or chemically equivalence nature of exchanges) of the exchange process. [16, 31] Analysis of the nature of the transition state clearly indicates that with respect to the positions of the nuclei responsible for the nuclear spin isomers, the two hydrogens are in perfectly symmetrically coordinated situation. [88–92] Moreover, the observed π-rotational symmetric (or mirroring replica) nature of the two hydrogens can be believed to be creating the necessary chemical equivalence situation for a possible quantum entanglement of the sates of the two water molecules participating in the double proton exchange process. [55] In light of this, we can say that at the site of Bjerrum D-site, where the quantum exchanges of states (through the exchanges of nuclear spin moments) are happening, the observed symmetric nature (or chemically equivalence nature) of the transition state can be believed to be responsible for a possible quantum entanglement of states of the two participating molecules. [88–92] To be noted here that compared to the non-planar cyclic dimer, in the transition state, the two hydrogens (being exchanged) are in one plane (fully planar arrangements). We emphasize on the fact that the possibility of quantum entanglement postulated here is purely qualitative viewpoints.

**Fig. 5:**
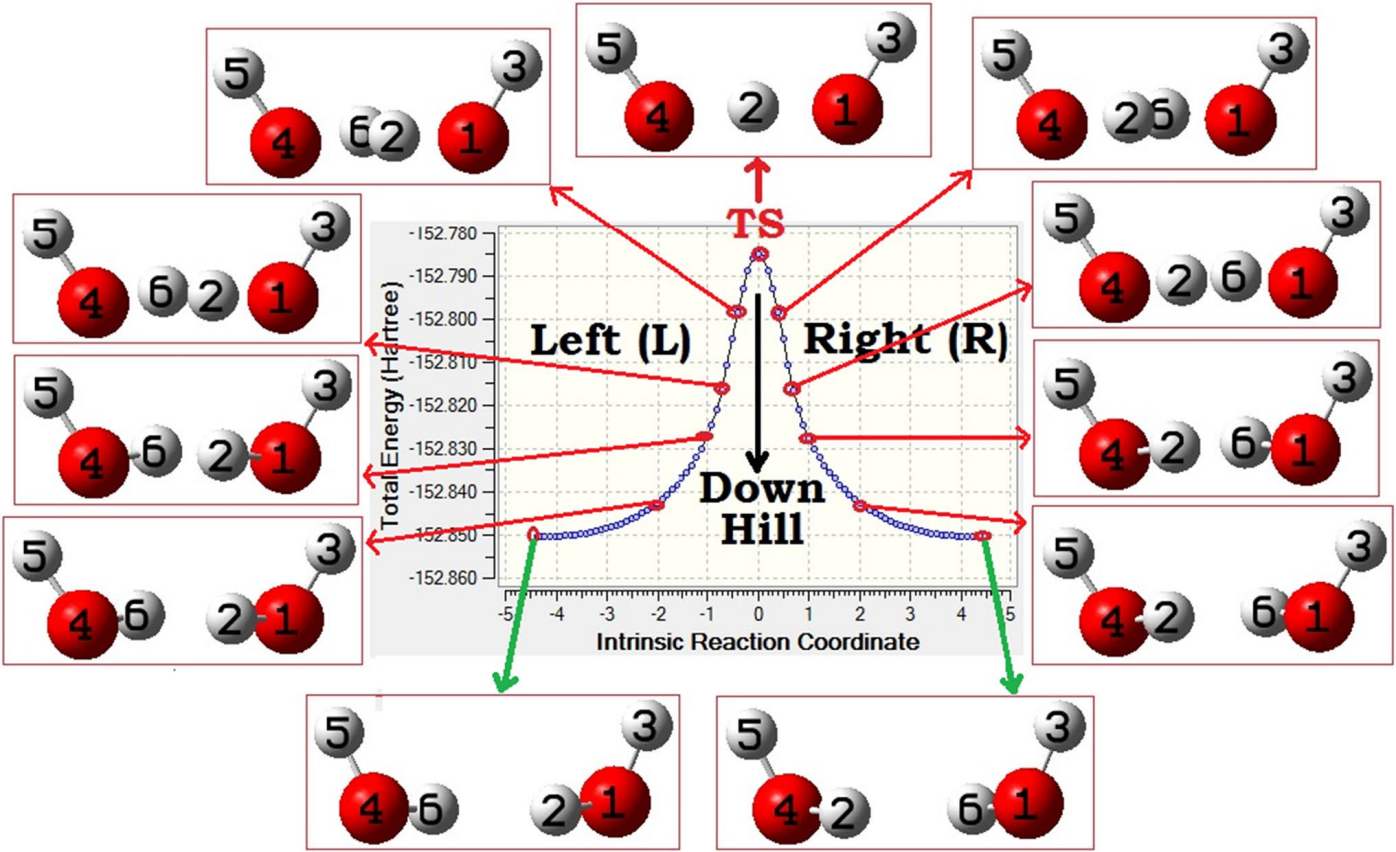
Mapped intrinsic reaction coordinates (IRC) paths for *cis-*TS, computed using B3LYP/6-31++G(d,p) methodology. Sequential structural changes moving downhill from the transition state are also shown from point-to-point (diagram is not to scale)

As discussed previously, at a Bjerrum D-defect site, besides the two hydrogen atoms participating in the proton exchanges, the remaining two free hydrogens are in alternative above and below the plane arrangements with respect to the two oxygen atoms of the water dimer (can be named as *trans*-TS: Fig. 4). Now, an alternative situation which can be imagined to be also possible, where the two free hydrogens, can be found above the plane containing two oxygen atoms (below the plane can also represent the same situation or indistinguishable situation). As such, a situation was not reported in any of the earlier works. Hence, we tried to locate such a replica transition state, by doing some rotational modification to the *trans*-TS (discussed above: Fig. 4) structure and using that as starting geometry for the transition state search. The obtained new transition state (can be named as *cis*-TS: vide infra) with IRC path is shown in Fig. 5.

Except the orientations of the two non-participating (or free) hydrogens, all other structural parameters of the *cis*-TS were found to be almost similar to the *trans*-TS (in *trans*-TS, the two free hydrogens were *trans* to each other, whereas in *cis*-TS, they were found to be *cis* to each other). Comparisons of the two important structural parameters show slightly enlarged values for the *cis*-TS than the *trans*-TS (1.248 Å and 1.212 Å for the *cis*-TS compared to 1.246 Å and 1.208 Å, respectively, for the *trans*-TS: refer Fig. 4). Like the *trans*-TS, a perfect mirroring type of entanglement and synchronization in relative motions of the two particles (hydrogens), participating in the dynamic double hydrogen transfer process, was observed. Also, from the symmetry perspective, like the *trans*-TS, clear indications of perfectly symmetrically coordinated situation, with respect to the positions of the nuclei responsible for the nuclear spin isomers, were observed. Also, like the *trans*-TS, in the *cis*-TS case also, we found that the relative motions of the two hydrogens are perfectly correlated, highly constrained, and exactly interlinked by one another.

### Homodromic natures of the transition states

**Fig. 6:**
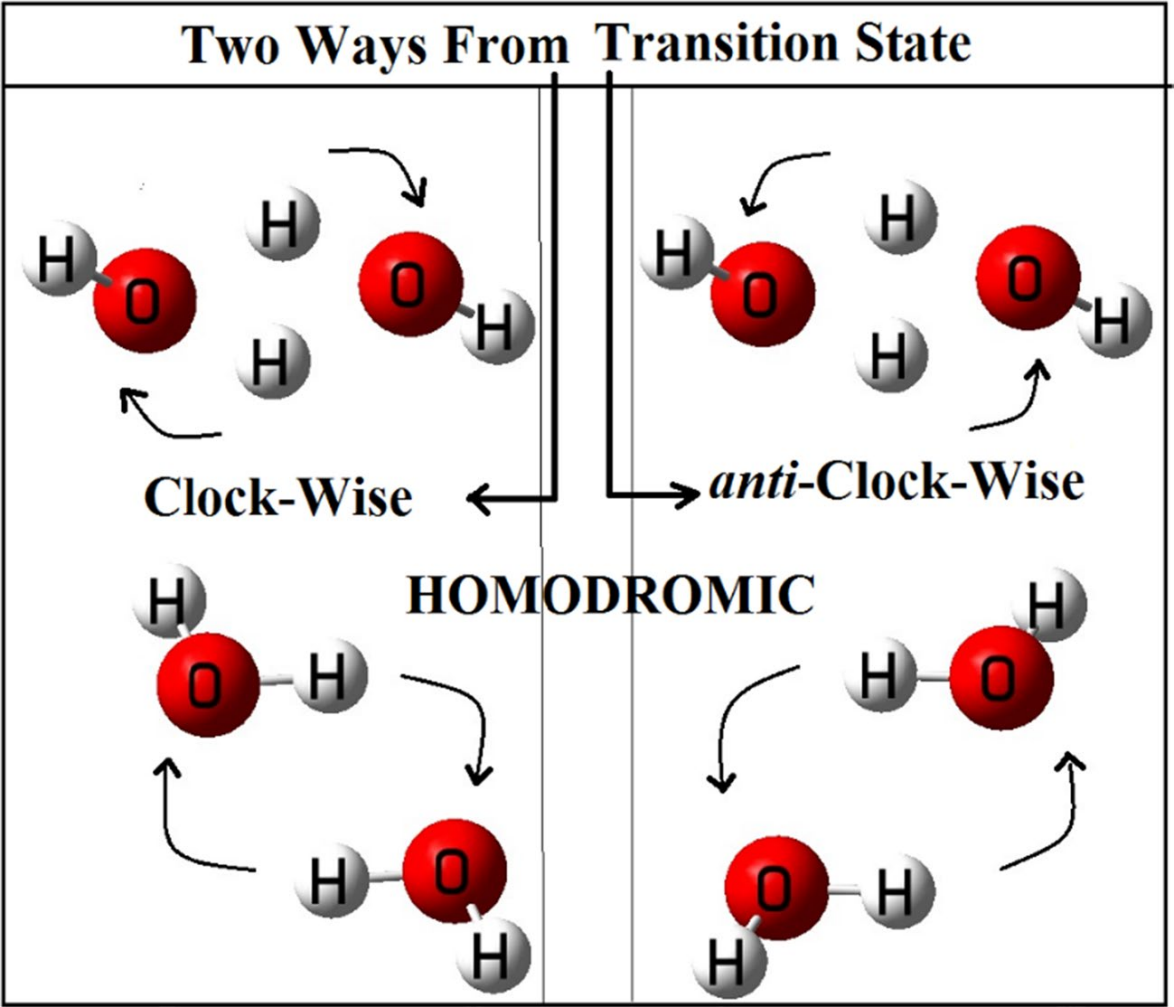
Pictorial representations of the homodromic natures of the quantum entangled dimer and transition state (*trans*-case). Geometries from B3LYP/6-31++G(d,p) methodology

Homodromic nature, which represents the pattern of a clockwise or anti-clockwise arrangements of the O-H bonds responsible for creating the network of hydrogen bonding in a water tetramer, has been reported recently. [93–96] In the case of the cyclic-non-planar water dimer discussed here, analysis of the two equivalent but non-mirror image replicas, exactly same homodromic nature, can be established. [93–96] But, for the first time, we have extended the homodromic principle to the replica transition state(s) discussed above. Pictorially, they are shown in Fig. 6. Like a homodromic state of a system, where the bonding array shows the clockwise or anti-clockwise movements, here in the replica transition state, homodromic nature can be attributed to the movements (either clockwise or anti-clockwise) of the two hydrogens participating in the exchange process. Though it is not mentioned anywhere, but we believe that similar homodromic nature with respect to the relative motions of the hydrogens in the transition state of the tetramer is definitely present in those earlier works. [93–96]

From the relative motions of the two hydrogens (for both the transition states), we observed that while moving downhills from the transition state, one way the motions are in a clockwise manner, on the other hand, in the other way, the relative motions are in an anti-clockwise manner. These behaviors shown by the spin isomeric cyclic water dimers and the linked transition states clearly support the existing homodromic natures. [93–96] Hence, we can say that, not only the tetramer but also in the cyclic dimer case, the homodromic principles are clearly applicable (not reported in all the earlier works related to water dimer). [93–96] Besides the homodromic nature, we can also guess that, while moving from one non-planar cyclic isomer to other, through the transition state, it passes from a non-Hermitian (or pseudo-Hermitian) to Hermitian type of situation.

### Effects of quantum entanglements

**Fig. 7:**
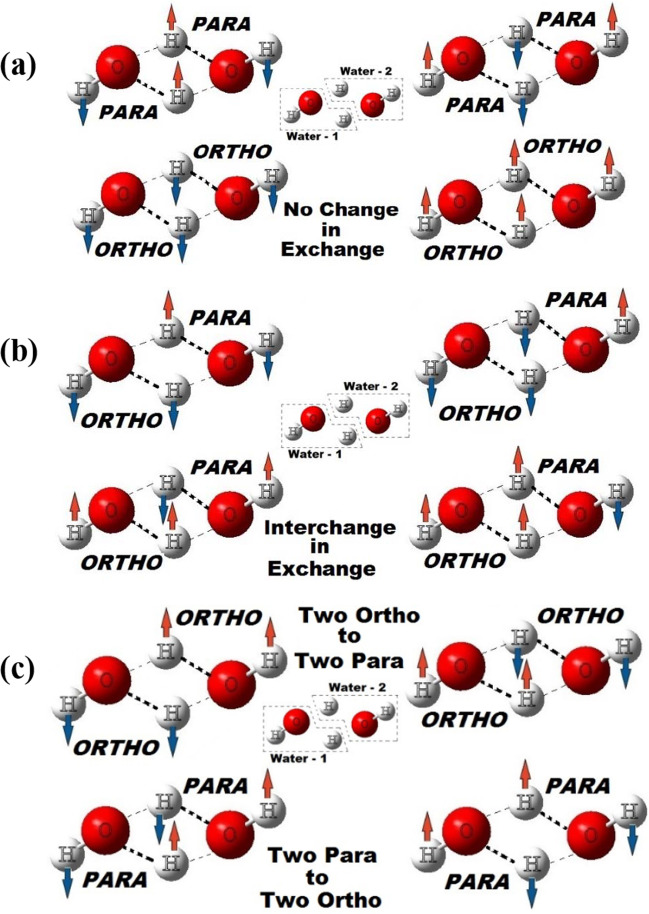
Pictorial representations of various possible ways of double proton exchanges, at a Bjerrum D-defect site, showing nuclear spin permutations between the two H_2_O molecules of the quantum entangled dimer. (a) no change in exchange, (b) interchange in exchange, (c) exchange and conversion. Geometries are from B3LYP/6-31++G(d,p) methodology

Though it is not discussed in any of the earlier reports, from a closer look at their reported transition states, we think that not only that Bjerrum D-type defect can facilitate a simultaneous dynamic double proton exchange but it can also help the double exchange to happen in a manner complaisant to each other, perfectly coordinated, and properly interlinked with each other, as if they are entangled. [55, 88–92] From the geometric point of view, the two hydrogen atoms participating in the exchange processes have the required π-rotational symmetric arrangements to render entanglement. [16, 31] At the site of Bjerrum D-defect, we analyzed all the possibilities of nuclear spin interactions (situations) that can happen at the above replica transition states (Figs. 4 and 5). All these possibilities which can basically be categorized into different and distinct types with respect to the spin arrangements are shown in Fig. 7 with appropriate spin representations. As shown in the Fig. 7 and discussed afterwards in this section, we restricted our discussions only for the *trans*-TS (Fig. 4) case. For the *cis*-TS (Fig. 5) case, exactly equivalent pictorial representations and arguments can be drawn like the case of *trans*-TS discussed below. Here, we discussed three different situations of spin exchanges between the entangled pairs of water molecules and tried to show how not all types of exchanges can lead to ortho-para interconversion. Tikhonov and Volkov also stated similar situation “The exchange without OP transitions, i.e., without change of energy (of resonance character), distinctly dominates” [4] might be the possible reason for the slow ortho-para interconversions.

#### No change in exchange

As shown in Fig. 7(a), with respect to the nuclear spins of the hydrogens (we represented blue arrows for spin-down cases and orange arrows for spin-up cases), we were able to establish four possible ways of spin interactions, for the double proton exchanges happening at a Bjerrum D-defect site. In these four cases, although the exchanges of hydrogens happen, but no overall changes can be observed for the spin isomers interchange (double proton exchanges have no effects on resultant ortho-para interconversion), hence, we categorized them as “no change in exchange.” These four cases can be distinctively sub-categorized further into two different ways, (1) ortho-ortho interactions with no spin-isomeric conversions and (2) para-para interactions with no spin-isomeric conversions. In both the cases, though the double hydrogen transfer happens, the transfer process restores the respective spin isomers back to their original spin isomeric identities. For the conversion prospective, these kinds of situations are not at all useful or completely ineffective. Instead, these circumstances can contribute to a slower conversion of ortho to para. As stated by Tikhonov and Volkov, “The exchange without OP transitions, i.e., without change of energy (of resonance character), distinctly dominates” [4] can axiomatically be found in the “no change in exchange” situations discussed here. From the entanglement prospective, in these situations, also the spin state of each participating molecule will be entangled. But, at this stage, we cannot say or access how the entanglement of spin states is going to affect the spin isomeric interconversions.

#### Interchange in exchange

From the Fig. 7(b), we were able to establish again four different possibilities, with respect to the nuclear spins of the hydrogens (blue arrows: spin-down and orange arrows: spin-up), of spin interactions, happening at a Bjerrum D-defect site. In these four cases, we have observed that during the double proton exchange process, although the interchanges between the spin isomers happen, but the net changes in the spin states before and after remaining conserved. Hence, they are categorized here as “interchange in exchange.” Unlike the previous case, no distinctive sub-categories can be visualized. In all the four cases, we can see that although the double hydrogen transfers are happening, but after the transfer processes, the net changes in the spin states are zero. Apparently, the observed no net change is possible since, one water molecule which was initially in ortho-state gets converted to para, and subsequently the other water molecule (counterpart) which was in para-state gets converted to ortho. Again, from the spin interconversion prospective, all the four cases will have overall zero effects on spin states interconversion. Such situations of “interchange in exchange” are like Tikhonov and Volkov proposal of “The exchange without OP transitions, i.e., without change of energy (of resonance character), distinctly dominates” [4]. Thus, these situations have the potentials to contribute to the observed slow rate for the ortho-para spin states interconversions. From the entanglement prospective, in these situations, also the spin state of each participating molecule will be entangled. But, at this stage, we cannot say or access how the entanglement of spin states is going to affect the spin isomeric interconversions.

#### Exchange and conversion

We were able to establish four situations with respect to the nuclear spins of the hydrogens, where possible spin exchanges and ortho-para conversions can happen at a Bjerrum D-defect site via the double proton exchange process. With the same conventions of representations (blue arrows and orange arrows representing the spin-down and spin-up cases respectively), all these four possibilities are shown in Fig. 7(c). These are the only four cases out of the twelve possibilities, which have potentials to affect the interchanges of the nuclear spin states and ortho-para interconversions. As shown for these four cases, in all these situations, ortho-to-para or para-to-ortho conversions are happening. Based on our observations, we have categorized them as “exchange and conversion,” which can further sub-categorized as (1) two ortho-states get converted to two para-states and (2) two para-states get converted to two ortho-states. Although these four states clearly represent the effective spin isomeric interconversion processes, but the two subcategories have potentials to affect either positively or negatively to the conversion rate based on their directionality.

Non-conserved nature of the overall spin (of individual H_2_O) before and after the double hydrogen transfer process, combined with the perfect entanglement of the hydrogen atoms in the replica transition state, can be expected to have effects on the ortho-para interconversion rates. [4] While in the situations representing the first case, two ortho-states of the water dimer get converted to para-states, on the other hand, in the situations representing the second case, two-para states of the water dimer get converted to ortho-states of the water dimer. From probabilistic point of view, both the cases have equal importance, equal opportunities, and equal credibility. On the other hand, equal probabilities of ortho-to-para and para-to-ortho conversions may be considered as one of the factors responsible for the slower conversion rate, as they are like opposing one another. Like the previous cases, from the entanglement prospective, in these situations, also the spin state of each participating molecule will be entangled. But, at this stage, we cannot say or access how the entanglement of spin states is going to affect the spin isomeric interconversions. In our view, ortho-para inter-conversion is not a continuous process, rather can happen serendipitously, with the many factors governing this simultaneously. Nevertheless, the four possible states shown in the Fig. 7(c) are the only available states for the possible spin entangled ortho-para spin isomeric interconversions.

## Conclusions

In this work, using quantum mechanical computations, we revisited the (H_2_O)_2_ dimer, only focusing on the non-planar cyclic dimers of water molecules. We have computed the two equivalent but non-mirror image isomers of the cyclic dimers, and we called them here as replica of each other. Comparison of the geometries obtained in our computations, we observed that they are very closely resemble with those of the recent experimental findings, of similar states of water dimers existing in the Bjerrum D-defect sites of ice. These recent experimental reports showed a linked transition state (only a *trans*-TS) between the two equivalent non-planar cyclic dimers of water (only trans-type dimer). A *cis*-TS and two equivalent *cis*-types of non-planar cyclic dimers of water molecules, reported in this work, have not been reported any earlier experimental or theoretical works (reported here for the first time). This analysis shows that for both the *cis*- and the *trans*- cases, the behaviors were found to be very similar. As the earlier experimental reports were showing only the *trans*-case (cyclic conformer and the TS); hence, we focused our discussions for the *trans-* case exclusively but mentioned that exactly similar behaviors can be drawn for *cis-* case also. Most of our discussions are from the structure related aspects, and the viewpoints mentioned here are mostly based on thorough analysis of all the possibilities. This work reports replica transition states facilitating simultaneous dynamic double hydrogen transfer processes. The observed natures of hydrogen transfer processes indicate that the motions of the two hydrogen atoms are well coordinated, well synchronized, and perfectly interrelated (correlated), and also at the same time, geometrically, the transition states were found to be π-rotational symmetric in natures. Based on these ideal situations, we argued for possibilities of quantum entanglements between the two water molecules at the replica transition states. We also argued for the existences of homodromic natures, both in the cyclic dimers and the transition states, an important aspect which was never discussed in earlier literature.

We analyzed many possible ways of interactions of spin states of the two water molecules present in the dimers and found that majority of them cannot support the ortho-para interconversions. We outlined only four situations, where ortho-para spin isomeric interconversions are possible, where two situations represent an ortho-to-para, and other two situations represent a para-to-ortho interconversions. Due to their different (or opposing) directionalities, even they are also capable of adversely affecting the ortho-para spin states interconversion rates. Out of 12 possible situations, only four (here also two cases are opposing the other two cases) are capable of bringing the interchange of nuclear spin states of water molecules. Such a situation unequivocally agrees with the proposal of Tikhonov and Volkov “The exchange without OP transitions, i.e., without change of energy (of resonance character), distinctly dominates” [4]. All these situations have the potentials to contribute to the observed slow rates for the ortho-para spin states interconversions. Based on our analysis of the non-planar cyclic dimer and its associated transition state, we have highlighted the possible roles of Bjerrum D-defect sites (present in the ice lattice due to the breaking of ice rules) in the dynamic hydrogen exchange processes responsible for the ortho-para spin states interconversions between two water molecules present in the water dimers. This work highlights the possible existences of quantum entanglements of the spin states of the water molecules, at the Bjerrum D-defect sites through the replica transition states. To be noted here that the possible quantum entanglement reported in this work is purely based on arguments of qualitative in natures, and at this stage, we cannot say or access the effects of entanglements on rates of spin isomeric interconversions. In conclusion, from a statistical point of view, many non-contributing exchange processes (the exchanges without any resultant ortho-para transitions), and in the contributing processes, the equal probabilities of ortho-to-para and para-to-ortho conversions (mutually opposing in directionality) can be considered as factors responsible for the slower conversion rates.

## Data Availability

Optimized coordinates of the isomers and transition states can be obtained from the author, through email request.
